# Pushing arterial-venous plasma biomarkers to new heights: A model for personalised redox metabolomics?

**DOI:** 10.1016/j.redox.2019.101113

**Published:** 2019-01-22

**Authors:** Andrew F. Cumpstey, Magdalena Minnion, Bernadette O. Fernandez, Monika Mikus-Lelinska, Kay Mitchell, Daniel S. Martin, Michael P.W. Grocott, Martin Feelisch, for the Xtreme Alps research group

**Affiliations:** a Critical Care Research Group, Southampton NIHR Biomedical Research Centre, Tremona Road, Southampton SO16 6YD, UK; b Anaesthesia and Critical Care Research Unit, University Hospital Southampton NHS Foundation Trust, Tremona Road, Southampton SO16 6YD, UK; c Integrative Physiology and Critical Illness Group, Clinical and Experimental Sciences, University of Southampton, Tremona Road, Southampton SO16 6YD, UK; d Clinical & Experimental Sciences, Faculty of Medicine, NIHR Southampton Biomedical Research Centre, University of Southampton and University Hospital Southampton NHS Foundation Trust, Tremona Road, Southampton SO16 6YD, UK; e Warwick Medical School, Division of Metabolic and Vascular Health, University of Warwick, Gibbet Hill Road, Coventry CV4 7AL, UK; f UCL Centre for Altitude, Space and Extreme Environment (CASE) Medicine, UCLH NIHR Biomedical Research Centre, Institute of Sport Exercise & Health, 170 Tottenham Court Road, London W1T 7HA, UK; g Intensive Care Unit, Royal Free Hospital, Pond Street, London NW3 2QG, UK; h Department of Anesthesiology, Duke University Medical School, NC, USA

**Keywords:** cGMP, cyclic guanosine monophosphate, CPET, cardiopulmonary exercise testing, EDTA, ethylenediaminetetraacetic acid, FRAP, ferric reducing ability of plasma, 4-HNE, 4-hydroxynonenal, H_2_S, hydrogen sulfide, 8-iso-PG, 8-isoprostaglandin, IC-MS, ion chromatography-mass spectrometry, LC-ESI-MS/MS, liquid chromatography-electrospray ionization-tandem mass spectrometry, NEM, N-ethylmaleimide, NO, nitric oxide, NO_2_^-^, nitrite, NO_3_^-^, nitrate, ONOO^-^, peroxynitrite, PaO_2_, arterial partial pressure of oxygen, PaCO_2_, arterial partial pressure of carbon dioxide, ROS, reactive oxygen species, RNS, reactive nitrogen species, RSI, reactive species interactome, RSNO, S-nitrosothiol, RSS, reactive sulfur species, RXNO, total nitroso species, SO_4_^2-^, sulfate, S_2_O_3_^2-^, thiosulfate, TFT, total free thiols, Thiols, Altitude, Hypoxia, Oxygen, Oxidative stress, Hydrogen sulfide

## Abstract

The chemical and functional interactions between Reactive Oxygen (ROS), Nitrogen (RNS) and Sulfur (RSS) species allow organisms to detect and respond to metabolic and environmental stressors, such as exercise and altitude exposure. Whether redox markers and constituents of this ‘Reactive Species Interactome’ (RSI) differ in concentration between arterial and venous blood is unknown. We hypothesised that such measurements may provide useful insight into metabolic/redox regulation at the whole-body level and would be consistent between individuals exposed to identical challenges. An exploratory study was performed during the *Xtreme Alps* expedition in 2010 in which four healthy individuals (2 male, 2 female) underwent paired arterial and central venous blood sampling before, during and after performance of a constant-work-rate cardiopulmonary exercise test, at sea level and again at 4559 m. Unexpectedly, plasma total free thiol and free cysteine concentrations remained substantially elevated at altitude throughout exercise with minimal arteriovenous gradients. Free sulfide concentrations changed only modestly upon combined altitude/exercise stress, whereas bound sulfide levels were lower at altitude than sea-level. No consistent signal indicative of the expected increased oxidative stress and nitrate→nitrite→NO reduction was observed with 4-hydroxynonenal, isoprostanes, nitrate, nitrite, nitroso species and cylic guanosine monophosphate. However, the observed arteriovenous concentration differences revealed a dynamic pattern of response that was unique to each participant. This novel redox metabolomic approach of obtaining quantifiable ‘metabolic signatures’ to a defined physiological challenge could potentially offer new avenues for personalised medicine.

## Highlights

• Exercise and high altitude (hypobaric hypoxia) significantly perturb redox balance.
• The role of thiols and reactive sulfur species in altitude acclimatization remains largely unknown.
• First measure of arteriovenous gradients of redox markers at altitude.
• Different individuals exposed to identical stresses display distinct redox response profiles.
• Redox metabolomics may offer new ways of personalizing medicine.

## 1 Introduction

Redox reactions constitute one of the most fundamental integrative regulatory systems that support human physiology, and our understanding of these systems has evolved at a remarkable rate in recent years. Redox reactions are intimately linked to both, oxidative stress and cellular bioenergetics. Over the past decade, the ‘oxidative stress’ concept (much of which initially revolved around cellular damage pathways associated with the formation of reactive oxygen species; ROS) evolved to become understood as a vital component of a wider ‘Redox Code’ that underpins most cellular signalling pathways and regulatory functions [20], [38], [39]. Beyond ROS, however, many other small reactive molecules are also involved in this regulation including reactive nitrogen species (RNS) and reactive sulfur species (RSS) [13], [14]. As well as controlling whole body metabolism, the chemical and functional interactions between these species (which we recently defined to constitute the ‘Reactive Species Interactome’; RSI [5]) allows organisms to sense and adapt to the many environmental stressors they experience throughout everyday life. We believe characterisation of these processes to be absolutely essential to arrive at a truly integrative understanding of human physiology in health and disease, although it is not clear at the moment what exactly we should measure to gauge the functional status of this RSI and its key regulatory nodes.

ROS, RNS and RSS are now emerging as equally important signalling molecules in their own right; targeting gene transcription, ion transport, metabolic activity and mitochondrial function [5], [14]. The ‘gasotransmitters’ nitric oxide (NO) and hydrogen sulfide (H_2_S) are the most prominent representatives of the RNS and RSS group of regulatory species, with peroxynitrite (ONOO^-^; [37]) and nitrosopersulfide (SSNO^-^; [6]) representing products of their chemical interaction with ROS [5]. The same protein targets that had been recognised as important players in the ROS/oxidative stress field earlier, cysteine redox switches, also seem to interact with RNS and RSS, leading to additional–sometimes similar but often distinct - downstream effects. The interaction of the cysteine thiol with RNS leads to formation of S-nitrosothiols (RSNO). S-nitros(yl)ation has long been recognised as prototypic post-translational redox modification [40], although the notion that RSNOs serve as important signalling entities has recently been challenged [36], [46]. By comparison, reaction of the same cysteine residues with RSS promotes formation of persulfide and polysulfide species. This sulfuration reaction, which is also known as S-sulfhydration in the biological literature dealing with H_2_S research [46], appears to be abundant and its products seem to have similarly widespread regulatory signalling functions [11], [17]. While the notion that sulfane sulfur is important for cell function is not new [43], this field is conceptually still in its infancy.

The tripeptide glutathione is known to be crucial for intracellular antioxidative defense by virtue of its reactive free cysteine thiol while serving as a cofactor for peroxidases and phase-II detoxification enzymes [28]. The cysteine required for its synthesis is transported, largely in the form of its disulfide, in the blood and taken up by cells via specific membrane transporters (e.g. the xCt antiporter system, in exchange for intracellular glutamate; [25]). In addition, homocysteine and cysteinylglycine are transported, in their free forms and as mixed disulfides, in blood. Circulating reduced, oxidized and protein-bound forms of these aminothiols have been defined to comprise the ‘plasma redox status’ [44], but relatively little is known about its regulation or the significance of individual constituents for extracellular redox status. More recently, the relative proportion of reduced and oxidized cysteine and glutathione in human plasma was found to be associated with the risk of death from coronary artery disease [35]. Unexpectedly, even the concentration of serum total free thiols was found to predict outcome in clinical conditions ranging from renal to cardiac disease [10], [22]; this may be due to the enhanced formation of cysteine and glutathione adducts with albumin under conditions of oxidative stress [5]. Even circulating sulfate, the final oxidation product of H_2_S and also a dietary constituent, may be of prognostic value in disease settings associated with oxidative stress [21]. In parallel with the renewed interest in the biological effects of these aminothiols and other sulfur-based mediators and biomarkers in biology, our group recently developed a novel mass spectrometry-based method that allows rapid and specific quantification of the plasma thiol metabolome with simultaneous measurement of aminothiols and sulfide [42].

Both, exercise and high altitude (hypobaric hypoxia) exposure represent two common but challenging physiological and metabolic stressors that are well recognised to result in significant perturbations in redox balance [19], [30], [45]. Historically, most studies have focused on how hypobaric hypoxia affects the production of ROS and RNS [22], while the role of RSS in altitude acclimatisation remains largely unknown. Moreover, as with most other clinical and physiological situations to date, previous studies have tended to limit examination of how the concentrations of redox system read-outs change to the peripheral venous circulation.

Recent work has shown that arterial and venous profiles of many metabolic markers, including those known to directly influence redox systems such as glutamate, differ in peripheral muscle compartments at sea level [18], and our own group has shown that tissue oxygen extraction remains relatively preserved at altitude despite oxygen uptake being limited [26]. The stable breakdown products that are generated as a result of formation and interaction of ROS, RNS and RSS with each other and their biological targets are usually not quantified in the arterial side of the circulation at altitude. To the best of our knowledge, the potential significance of any arteriovenous concentration differences in redox thiol status, in particular that of sulfide-related species, has not been explored.

We hypothesised that significant differences between arterial and venous plasma concentrations would be seen for many redox markers during exercise, and that these differences would be further exacerbated at altitude. We further expected that, while magnitudes of responses might differ between individuals, the overall direction of the responses would be consistent in participants exposed to identical challenges.

## 2 Methods

Full details of the conduct of the Xtreme Alps 2010 expedition have been published elsewhere [7], [27]. The work presented here was part of a small pilot sub-study performed alongside the main Xtreme Alps research project. All participants gave written informed consent, and ethical approval was received from the research ethics committees at both University College London, UK and the University of Turin, Italy.

### 2.1 Overall study design

Four participants (2 male, 2 female) of the laboratory team, not enrolled as study subjects in the main Xtreme Alps 2010 study, underwent paired arterial and venous blood sampling before, during and after performance of a constant work rate cardiopulmonary exercise test (CPET) at near sea-level in London, UK (75 m), and again at the Margherita Hut high altitude research laboratory (4559 m).

### 2.2 Ascent profile

Following air travel to Milan, Italy, and immediately after transfer by coach to Alagna (1200 m), all participants ascended by cable car to Punta Indren (3200 m) and then walked to the Gnifetti Hut (3647 m). Subjects continued to climb on foot to the Margherita Hut (4559 m) after spending 2 nights at 3647 m. Measurements for this study protocol were taken on days 5 and 6 at 4559 m (i.e. days 7 and 8 at altitude). By this time every subject was well acclimatized, and no evidence of acute altitude illness or any other contra-indication to exercise testing was present. Mean barometric pressure during testing periods at sea level and altitude were 101.7 kPa and 58.1 kPa respectively.

### 2.3 Exercise testing

Exercise tests were performed on an electronically braked cycle ergometer exercise bike (Lode Corival; Lode, Groningen, Netherlands) and breath-by-breath analysis of inspired/expired oxygen and carbon dioxide was achieved using a cardiopulmonary exercise testing system (Metamax 3b; Cortex, Leipzig, Germany). This system has been previously validated at high altitude and used successfully at altitudes up to almost 8000 m by our group [24]. All subjects properly fasted before commencing the test and maintained a pedalling cadence of 60 rpm throughout the exercise protocol.

**Fig. 1:**
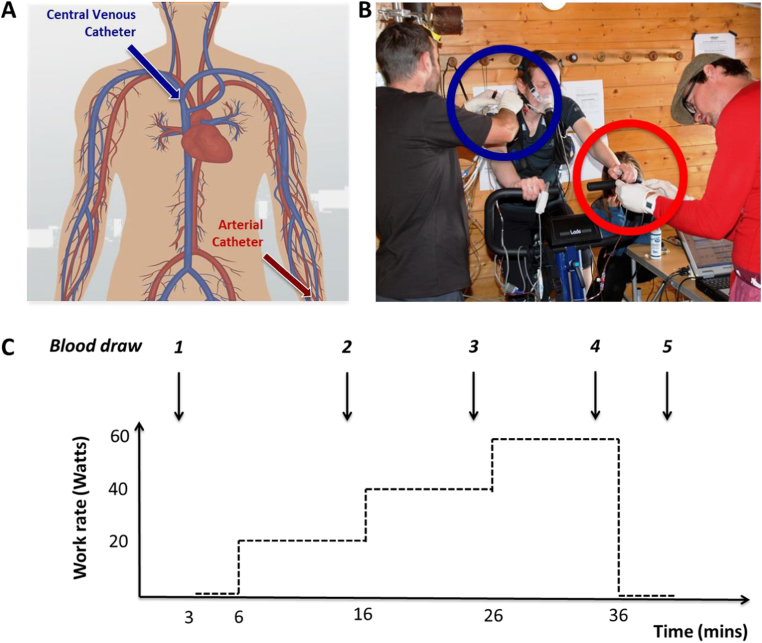
Blood sampling points (A) with approximate positions of central venous and radial artery catheters; simultaneous drawing of arterial (red circle) and venous blood (blue circle) during exercise at high altitude (B); and exercise protocol with time points at which blood was collected for immediate pH/blood gas and later biomarker analysis (C). See Methods for details. (For interpretation of the references to color in this figure legend, the reader is referred to the web version of this article).

After sitting stationary on the exercise bike for 3 min (‘rest’) and a further 3 min of unloaded cycling, subjects cycled at three steady-state work rates (20, 40 and 60 W) for 10 min each, before stopping the exercise (see Fig. 1). These work rates were selected to target the exercise intensity of participants cycling to be below their anaerobic threshold at sea-level and at altitude, based on our group's previous exercise studies at altitude [12], [24], [27]. At altitude all subjects had an extra 3 min of rest. Physiological observations were recorded throughout each test using a non-invasive blood pressure cuff on the upper arm and a pulse oximeter placed on the right index finger.

### 2.4 Catheter placement and blood sampling

Immediately before each exercise test, an experienced anaesthetist and/or intensivist inserted a central venous catheter into each subject's right internal jugular vein (using a standard Seldinger technique under ultrasound guidance) and placed an arterial catheter into the non-dominant radial artery.

Paired central venous and arterial blood samples were collected from these catheters simultaneously by 2 investigators in the last minute of the rest period (time point 1), 8 min into each constant work rate period (time points 2, 3 and 4, respectively) and 3 min after stopping exercising (time point 5), as illustrated in Fig. 1. pH, arterial partial pressure of oxygen (PaO_2_) and arterial partial pressure of carbon dioxide (PaCO_2_) readings from each sample were recorded immediately using a Siemens blood gas analyser (validated and used at similar altitudes in previous studies by our group) [15], [24].

### 2.5 Blood processing and analysis

Separate aliquots were collected with EDTA as anticoagulant right after those harvested for blood gas analyses, immediately subjected to centrifugation to obtain plasma, aliquoted at altitude and kept frozen at − 40 °C (both at sea level and at high altitude) before transfer on dry ice into a − 80 °C freezer for long-term storage. Final biochemical sample analysis was carried out in Southampton, UK. Plasma biomarker concentrations were quantified in batches of no more than 20 samples in a staggered fashion; samples for NO metabolite and thiol metabolome analysis were pretreated with N-ethylmaleimide (NEM) whereas those for other biomarkers were used immediately after thawing without further derivatization. For the former, frozen plasma aliquots were thawed in the presence of an excess of NEM in PBS to achieve a final concentration of 10 mM; this was accomplished by adding a 100 mM NEM stock in 100 mM ammonium phosphate (pH 7.40) at a 1:10 (v:v) ratio to a sample aliquot immediately after thawing, followed by vortexing. Daily calibrations with authentic standards were performed for all biochemical assays (and all glassware, collection tubes and pipettes were rinsed with ultrapure water to reduce background contaminant levels of nitrite and nitrate). Analyses were performed at least in duplicate and values averaged, except for nitrite, nitrate, sulfate, thiosulfate and RXNO where only a single determination was performed due to sample volume limitations.

Commercially available ELISA/EIA assays were used to quantify the concentrations of 4-hydroxynonenal (4-HNE; STA-838; Cell Biolabs San Diego, USA), 8-isoprostane (8-IsoPG; 516630; Cayman Chemicals, Ann Arbor, Michigan, USA) and cyclic GMP (cGMP; KGE003; R & D Systems, Minneapolis, USA), and all kits performed in accordance with the manufacturer's instructions and protocols. Nitrate (NO_3_^-^) and nitrite (NO_2_^-^) concentrations were quantified using a dedicated high-performance liquid chromatography analyser (ENO20, Eicom) following sample deproteinisation by precipitation with methanol and centrifugation at 16,000 ×*g* for 20 min. Concentrations of total nitrosation products (RXNO) were measured by gas-phase chemiluminescence of bound NO following reductive denitrosation. Nitrite was removed from NEM-treated EDTA plasma by reaction with acidic sulfanilamide prior to analysis, followed by injection of treated aliquots into an acidic triiodide-containing reduction chamber, and the amount of NO liberated from low-molecular weight and protein nitroso-species was quantified by a gas-phase chemiluminesence analyser (CLD 77 am sp, EcoPhysics), as previously described [9], [23]. Total “antioxidant power” was measured by the Ferric Reducing Activity of Plasma (FRAP) assay [2]. The concentrations of total free thiols (TFTs) in plasma were determined spectrophotometrically using Ellman's reagent [22]. Plasma concentrations of sulfate (SO_4_^2-^) and thiosulfate (S_2_O_3_^2-^) were quantified after separation on a Dionex IonPac AS11-HC-4 µm column and detected as the monoprotonated species using ion chromatography mass spectrometry (IC-MS; Dionex ICS-5000 + system, Thermo Scientific). Ultrahigh-performance liquid chromatography in combination with electrospray-ionization tandem mass spectrometry (LC-ESI-MS/MS) was used to separate and quantify all of the remaining analytes including sulfide and biological aminothiols such as cysteine, N-acetylcysteine, homocysteine, glutathione, cysteinylglycine, and glutamylcysteine in the form of NEM-adducts as described in detail by our group elsewhere [42]. In addition to the free thiols, their total concentrations (free + protein-bound forms and disulfides) were determined after sample pre-processing with dithiothreitol (DTT). For this purpose, already NEM-reacted sample aliquots were subjected to reduction by addition of an excess of DTT at room temperature, as described in detail elsewhere [42]. This procedure works even with NEM-pretreated samples (as DTT also reacts with this alkylans), provided the excess NEM is first neutralized by an adequate concentration of DTT (beyond what is required for reduction of bound thiols after NEM neutralization), followed by subsequent incubation for 30 min at room temperature to achieve complete reduction and addition of another excess of NEM for derivatization of the liberated thiols [42]; appropriate dilutions in 10 mM ammonium phosphate buffer were made before subsequent analysis by LC-MS/MS. Because plasma samples were not treated with NEM immediately after centrifugation, the oxidized form are artificially elevated, preventing us from calculating accurate redox ratios; therefore, concentrations of oxidized thiols are not reported.

### 2.6 Statistical analysis

Physiological observational and blood gas data were all treated as non-parametric, and results are therefore presented as median (range) for these values. For blood gas data, significance was assumed if p < 0.05 using the Wilcoxon Signed-Rank Test. Results of plasma biomarker analyses are presented as mean ± SD in tables whereas, for the sake of clarity of presentation, only the means are displayed in the figures. All calculations and figures were produced using GraphPad Prism 7.0.

## 3 Results

### 3.1 Exercise test performance and sample collection

All 4 subjects reached 4559 m and remained at this altitude for the full duration of the expedition. One subject was initially unwell at 4559 m, developing symptoms of acute mountain sickness shortly after arrival at 4559 m, but these had fully resolved before commencing the exercise protocol. All venous and arterial catheters were successfully inserted on the first attempt without any complications. Similarly, all exercise tests at sea level and at altitude were completed successfully without any problems; moreover, all samples were collected according to plan and either analysed immediately (pH, blood gases) or processed and frozen (for later analysis of biomarkers) as above.

Normal physiological responses to exercise were observed throughout each test. Participants’ blood pressure and heart rate both increased at higher exercise work rates, whilst peripheral oxygen saturations decreased (see Table 1). One blood pressure reading could not be recorded at 60 W due to erratic readings during that level of exertion in this subject.

**Table 1:** Study participants’ (P1, P3 male; P2, P4 female) blood pressure (BP; mmHg), heart rate (HR; beat per minute, bpm) and peripheral oxygen saturation (SpO_2_; %) recorded at rest (T1), during exercise (T2-T4) and in early recovery (T5), at sea level and at altitude.

### 3.2 Blood gas analyses

Arterial pH decreased from 7.44 (7.43–7.46) at rest to 7.43 (7.42–7.44) at 60 W at sea level. Similarly, the 60 W work rate at 4559 m was associated with a decrease in arterial pH from 7.49 (7.45–7.52) at rest to 7.45 (7.43–7.48). Resting PaO_2_ significantly decreased from 12.7 (11.4–13.2) kPa at sea level to 6.8 (6.3–7.0) kPa with exposure to the hypobaric hypoxia at 4559 m altitude (p < 0.001). Resting PaCO_2_ also significantly decreased from 5.6 (5.3–6.4) kPa at sea level, to 3.3 (3.1–3.8) kPa at 4559 m (p < 0.001).

### 3.3 Plasma biomarkers

**Fig. 2:**
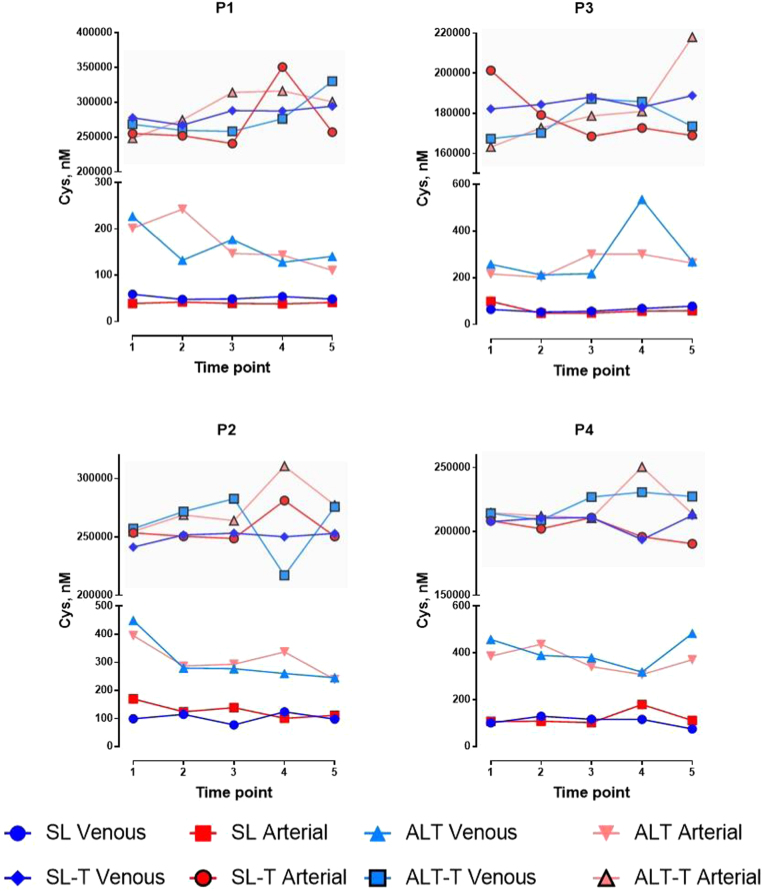
Steady-state arterial (red) and venous (blue) concentrations of free and total (T; shaded area) cysteine in study participants’ (P1, P3 male; P2, P4 female) plasma at rest T1, T2–T4, and T5, during exercise [2], [3], [4] and in early recovery [5], at sea level (SL, circles and squares) and at high altitude (ALT–light triangles). (For interpretation of the references to color in this figure legend, the reader is referred to the web version of this article).

**Fig. 3:**
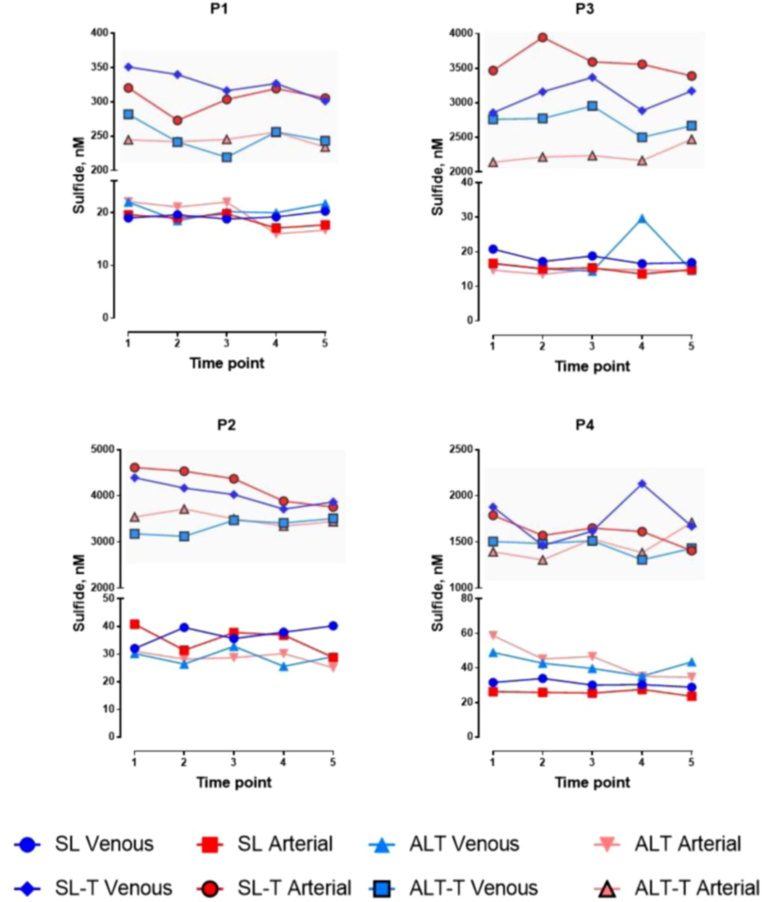
Steady-state arterial (red) and venous (blue) concentrations of free and total (T; shaded area) sulfide in study participants’ (P1, P3 male; P2, P4 female) plasma during rest [1], exercise [2], [3], [4] and early recovery [5] at sea level (SL, circles and squares) and at high altitude (ALT–light triangles). (For interpretation of the references to color in this figure legend, the reader is referred to the web version of this article).

**Fig. 4:**
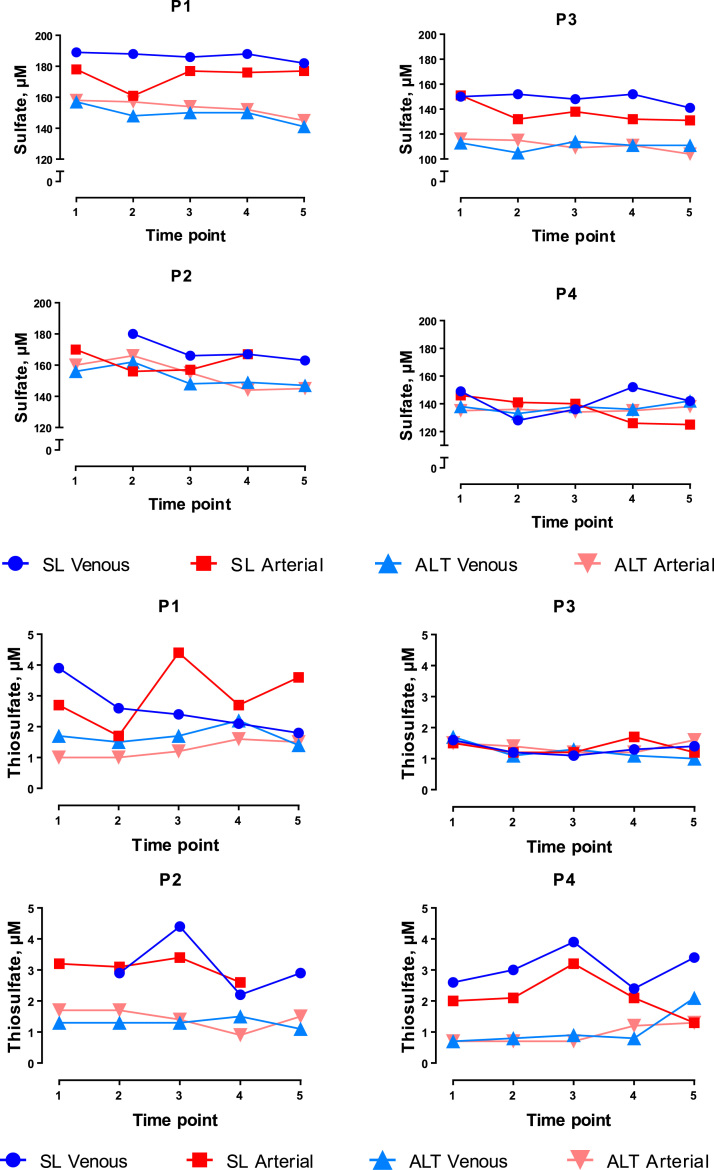
Arterial (red) and venous (blue) steady-state concentrations of sulfate and thiosulfate in study participants (P1, P3 male, P2, P4 female) at rest (T1), during exercise (T2–4) and in early recovery (T5) at sea level (SL; circles and squares) and at high altitude (ALT–light triangles). Missing data points were due to insufficient sample volumes available. (For interpretation of the references to color in this figure legend, the reader is referred to the web version of this article).

Changes in total free thiols (TFT) and cysteine levels are shown in Table 2 (and graphically in Supplementary Figure 6) and Fig. 2, respectively, with levels of both markedly increasing under hypoxic conditions at altitude in all subjects. These changes persisted and remained relatively unchanged throughout all levels of exercise with minimal arteriovenous differences during this observation period. By contrast, circulating sulfate and thiosulfate concentrations (Fig. 4) were lower upon exposure to hypobaric hypoxia. Despite this, only relatively minor changes to circulating sulfide levels were seen throughout exercise, both at sea level and at altitude (Fig. 3). As previously reported for healthy volunteers residing at sea level [42], concentrations of bound cysteine and sulfide exceeded free levels by orders of magnitude (Fig. 2, Fig. 3). No consistent differences in free/total N-acetylcysteine, homocysteine, glutathione, cysteinylglycine and glutamylcysteine were observed between sea level and altitude (see Supplementary Figures 1–5).

**Table 2:** Concentrations of study participants’ (P1, P3 male; P2, P4 female) circulating total free thiols (TFT) and total antioxidants (Ferric Reducing Ability of Plasma assay, FRAP) recorded at rest (T1), during exercise (T2-T4) and in early recovery (T5), at sea level and at altitude.

**Fig. 5:**
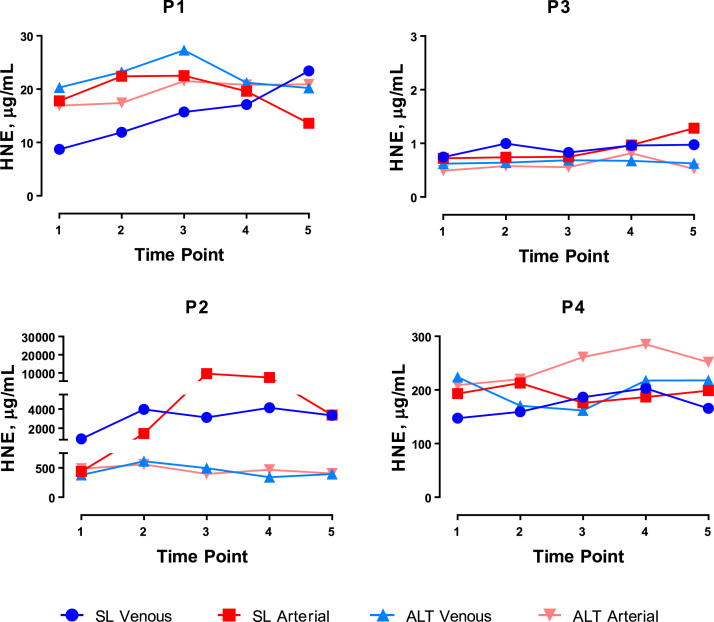
Arterial (red) and venous (blue) steady-state plasma concentrations of 4-hydroxynonenal (HNE) in study participants (P1, P3 male; P2, P4 female) at rest (T1), during exercise (T2-T4) and in early recovery (T5), at sea level (SL; circles and squares) and at high altitude (ALT; light triangles). (For interpretation of the references to color in this figure legend, the reader is referred to the web version of this article).

**Fig. 6:**
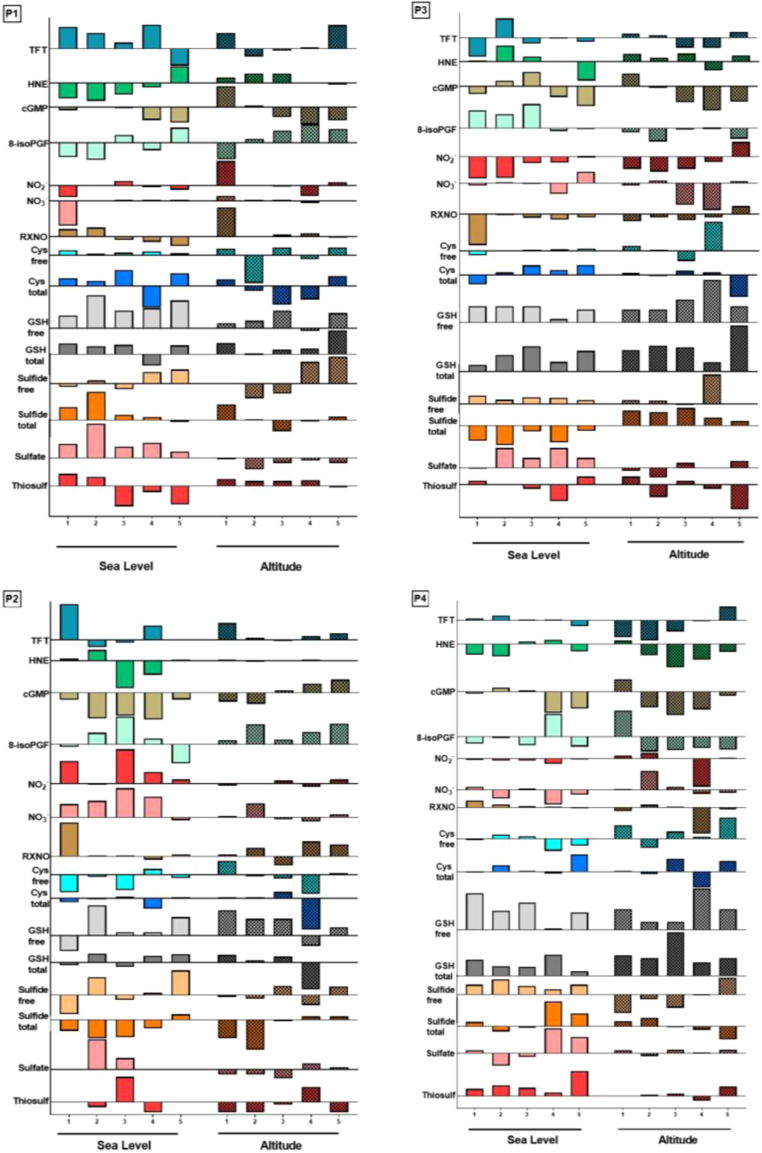
Pattern of responses to the physiological stress of skeletal muscle activity (bicycle exercise) at sea level and high altitude. Exemplary differences in absolute steady-state concentrations of various redox markers in arterial and venous plasma during rest (T1), exercise (T2-T4) and in early recovery (T5) in study participants (P1, P3 male; P2, P4 female) are plotted for test results obtained at normoxia (sea level) and during chronic hypoxia (altitude). Values above the horizontal bar denote higher venous than arterial concentrations and vice versa (units omitted for sake of clarity).

Results of nitrate (NO_3_^-^), nitrite (NO_2_^-^), total nitroso species (RXNO) and cyclic guanosine monophosphate (cGMP) measurements are shown in Table 3. Only rather moderate differences were observed in total plasma antioxidant power (FRAP; Table 2) and oxidative stress markers such as 4-hydroxynonenal (HNE) and 8-isoprostanes, which are shown in Fig. 5 and Table 4, respectively. No consistent signal indicative of elevated oxidative stress was apparent at high altitude, nor was there an indication for the expected nitrate and/or nitrite reduction in hypoxia (or an increased NO bioavailability). Measurements of each of these markers varied greatly between different participants, in both arterial and venous compartments, and at each stage of exercise. However, when all of the biomarkers measured were combined and plotted as arteriovenous concentration differences for each sampling condition (see Fig. 6), distinct redox profiles emerged that suggested substantial differences in dynamic responses of individual contributors to the RSI. When expressed this way, each participant demonstrated a unique response pattern to the metabolic stress of exercise under normoxic and hypoxic conditions.

**Table 3:** Plasma concentrations of study participants’ (P1, P3 male; P2, P4 female) nitric oxide related biomarkers including nitrate, nitrite, nitroso species (RXNO) and cyclic guanosine monophosphate (cGMP) recorded during rest, exercise and early recovery at sea level and at high altitude.

**Table 4:** Study participants’ (P1, P3 male; P2, P4 female) circulating 8-isoprostane (8-isoPG) concentrations in arterial and venous plasma at rest (T1), during exercise (T2-T4) and in early recovery (T5), at sea level and at altitude.

## 4 Discussion

The results from our present study demonstrate that exposure to hypobaric hypoxia is accompanied by markedly higher concentrations of circulating cysteine and protein free thiols yet lower concentrations of sulfate and thiosulfate, with only moderate changes in other aminothiols and sulfide. In addition, the concentration of various other constituents of the RSI differed between the arterial and venous side of the circulation, indicative of either net consumption or production of these substances or their precursors upon passage through body organs. Importantly, although the nature of the metabolic (exercise) and environmental (hypobaric hypoxia at altitude) stresses experienced by each participant were identical, the magnitude and directionality of the responses to these combined stressors markedly differed between individuals. In particular, the bound thiol pool (including sulfide) showed a surprising dynamic that may be used to characterize the redox metabolomic phenotype of individuals and perhaps serve as a useful tool in personalizing medicine in the future.

We believe this study to be the first in reporting field measurements of RSS and arteriovenous gradients of redox biomarkers at high altitude. The challenges of performing invasive field studies at altitude are substantial and frequently limit sample sizes in such studies; nevertheless, results of other (identically powered) altitude studies have demonstrated interesting findings with the potential for significant impact [15], [26]. Our current study now demonstrates that arteriovenous gradients of plasma biomarkers can safely be determined at altitude without complications. Central venous catheters were chosen rather than full pulmonary artery catheters because of the increased number of adverse events associated with pulmonary artery catheter insertion; however, using a relatively low insertion approach in the right internal jugular vein under ultrasound guidance, all catheters should have sat in the superior vena cava giving access to blood that reflects true central venous values [26].

We have demonstrated robust elevations in TFT concentrations at altitude throughout all stages of exercise and although the significance of this response is uncertain, such a marked and consistent response across all subjects suggests a tightly regulated response to hypoxia. This result was unexpected and contrary to our hypothesis that circulating free thiol levels would be lower under conditions of chronically elevated oxidative stress, which typically prevails at high altitude [19], [23]. Other studies have suggested increased TFT concentrations are associated with improved cardiovascular outcomes and patient survival [10], [22], possibly by acting as antioxidant ‘buffer’ to ROS and reducing harmful oxidative stress [4]. Plant studies have also suggested thiol concentrations increase at moderate altitudes in lichens to confer protection against increased free radical formation [16]. Similarly, intracellular protein thiols have been demonstrated to sense and regulate cellular responses to hypoxia, including regulatory cysteines within the Na^+^,K^+^-ATPase that are thought to confer this critical enzyme's ability to sense oxygen levels [3]. Thiol availability may also alter production of other reactive species, particularly NO [32], allowing further signalling crosstalk in response to hypoxic stress. What accounts for the higher TFT concentrations at altitude warrants further investigation, but a likely possibility is enhanced cleavage of circulating mixed disulfides by extracellular or outer membrane reductases (such as protein disulfide isomerase and other members of the thioredoxin family; [29], [33]). This may, in part, be captured by the unspecific “antioxidant power” assay, FRAP (see Table 2). Consistent with literature data, documenting a higher activity for men compared to women [1], FRAP was robustly elevated at altitude only in the two male participants. Since S-cysteinylated albumin is an established marker of oxidative stress [34] and residence at altitude is typically associated with higher oxidative stress, such a mechanism would also account for the elevated free cysteine levels observed at altitude. Remarkably, circulating free sulfide levels did not change with altitude although the concentrations of its oxidation products, thiosulfate and sulfate were lower than at sea level. These observations are consistent with alterations in flux through the transsulfuration pathway, which is known to be redox-sensitive [31].

Changes in other biomarker concentrations in response to these stressors were much more variable, for example 4-HNE (the protein adduct of a reactive lipid oxidation product) concentrations differed by several orders of magnitude between individuals, both at baseline and during exercise. Reasons for these differences are likely multifactorial while still consistent with previous studies.

Levels of nitrate in this study were similar to those reported previously by our group in other cohorts sojourning at similar altitudes in Nepal [22], while levels of nitrite were much lower in this group albeit still similar to levels reported by others [8]. This could be due to different dietary intakes by these different groups–everyone on the Xtreme Alps expedition (including those participating in this study) consumed a low nitrate diet as an important dietary element of the expedition's main study [7], [27]. Venous cGMP concentrations were also similar to previous measurements in Nepal [23]. Interestingly though, arterial concentrations were generally higher in this cohort, with the subsequent arteriovenous gradient perhaps suggesting that the increased NO availability seen at altitude is more predominant in the preferentially well-oxygenated arterial side of the circulation.

Measurement of plasma biomarker arteriovenous gradients has previously been proposed as a more sensitive way of detecting metabolic changes in individuals, and–by choosing from which artery and vein blood is sampled - could also allow a relatively simple way of monitoring organ specific changes in different disease states [18]. The A/V concentration differences observed using the approach applied in the current study are a reflection of whole-body metabolism, which–taking flow rates into account–should also allow to calculate uptake/consumption and/or production rates. Although ‘omic’ study approaches have recently started being applied to the altitude environment [41], the additional information that arteriovenous gradients may provide in the context of metabolomic approaches at altitude have never been explored before. Furthermore, the lack of any apparent increase in tissue oxygen extraction at altitude suggests that adaptations occur on a cellular level to improve tissue oxygen handling in this environment [26]. These may include subtle redox-related metabolic changes that might not be detectable otherwise.

Our study has strengths and limitations. Its strength relates to the relatively large number of analytes assessed in both arterial and venous plasma samples, in a longitudinal fashion during graded metabolic stress, both at sea level and at high altitude. However, our study is essentially preliminary as samples were only obtained from four individuals. A clear limitation is the long duration between collection and analytical analysis of the samples (approximately 8 years) during which time the concentration of some analytes may have changed in the frozen state. This is particularly relevant for free thiols in the absence of NEM; thus free thiol concentrations reported in this exploratory study should not be compared to literature values as they do not reflect physiological concentrations. However, the analytical method used in the present study [42] was not available at the time of sample collection and all samples were treated in an identical fashion.

Apart from the altitude setting, redox metabolomics appears to offer new ways of targeting and personalizing medicine. These results show that small molecules important to whole-body redox regulation appear have very distinct response patterns in different individuals exposed to identical stressors such that each participant revealed his/her unique metabolic signature. The implications of this finding remain to be explored in detail, but may potentially be far-reaching–in particular, if it allowed personalised metabolic phenotyping based on the (epi)genetic make-up and exposome experience of an individual. In the future, a more refined and deeper understanding of exactly how these stress response pathways work together as part of the RSI could offer new ways of detecting, monitoring and treating different diseases; it may even be of use in characterizing the pharmacological action of drugs in vivo.
