# Supplementary material for: Pushing arterial-venous plasma biomarkers to new heights: A model for personalised redox metabolomics?
Source: Redox Biol. 2019 Jan 22;21:101113. doi: 10.1016/j.redox.2019.101113 (PMC6369731; doi:10.1016/j.redox.2019.101113)
Supplement: Supplementary file 1 — Supplementary material [file mmc1.docx]

**Supplementary Information**

for article

**Pushing Arterial-Venous Plasma Biomarkers to New Heights:**

**A Model for Personalised Redox Metabolomics?**

Andrew F Cumpstey^1-3^, Magdalena Minnion^4^, Bernadette O Fernandez^4,5^, Monika Mikus-Lelinska^4^, Kay Mitchell^1-3^, Daniel S Martin^6,7^, Michael P W Grocott^1-3,8^ and *Martin Feelisch^3-5^ for the Xtreme Alps research group.

1. Critical Care Research Group, Southampton NIHR Biomedical Research Centre, Tremona Road, Southampton, SO16 6YD, UK
2. Anaesthesia and Critical Care Research Unit, University Hospital Southampton NHS Foundation Trust, Tremona Road, Southampton, SO16 6YD UK
3. Integrative Physiology and Critical Illness Group, Clinical and Experimental Sciences, University of Southampton, Tremona Road, Southampton, SO16 6YD UK
4. Clinical & Experimental Sciences, Faculty of Medicine, NIHR Southampton Biomedical Research Centre, University of Southampton and University Hospital Southampton NHS Foundation Trust, Tremona Road, Southampton, SO16 6YD UK.
5. Warwick Medical School, Division of Metabolic and Vascular Health, University of Warwick, Gibbet Hill Road, Coventry CV4 7AL, UK
6. UCL Centre for Altitude, Space and Extreme Environment (CASE) Medicine, UCLH NIHR Biomedical Research Centre, Institute of Sport Exercise & Health, 170 Tottenham Court Road, London, W1T 7HA, UK
7. Intensive Care Unit, Royal Free Hospital, Pond Street, London, NW3 2QG, UK
8. Department of Anesthesiology, Duke University Medical School, North Carolina, USA

*Correspondence to:*

Martin Feelisch ([m.feelisch@soton.ac.uk](mailto:m.feelisch@soton.ac.uk))

**Suppl. Fig.1:** Arterial (red) and venous (blue) steady-state concentrations of free reduced and total (T) glutathione (GSH) in study participants (P1, P3 male, P2, P4 female) at rest (T1), during exercise (T2-4) and in early recovery (T5) at sea level (SL; circles and squares) and at high altitude (ALT – light triangles).


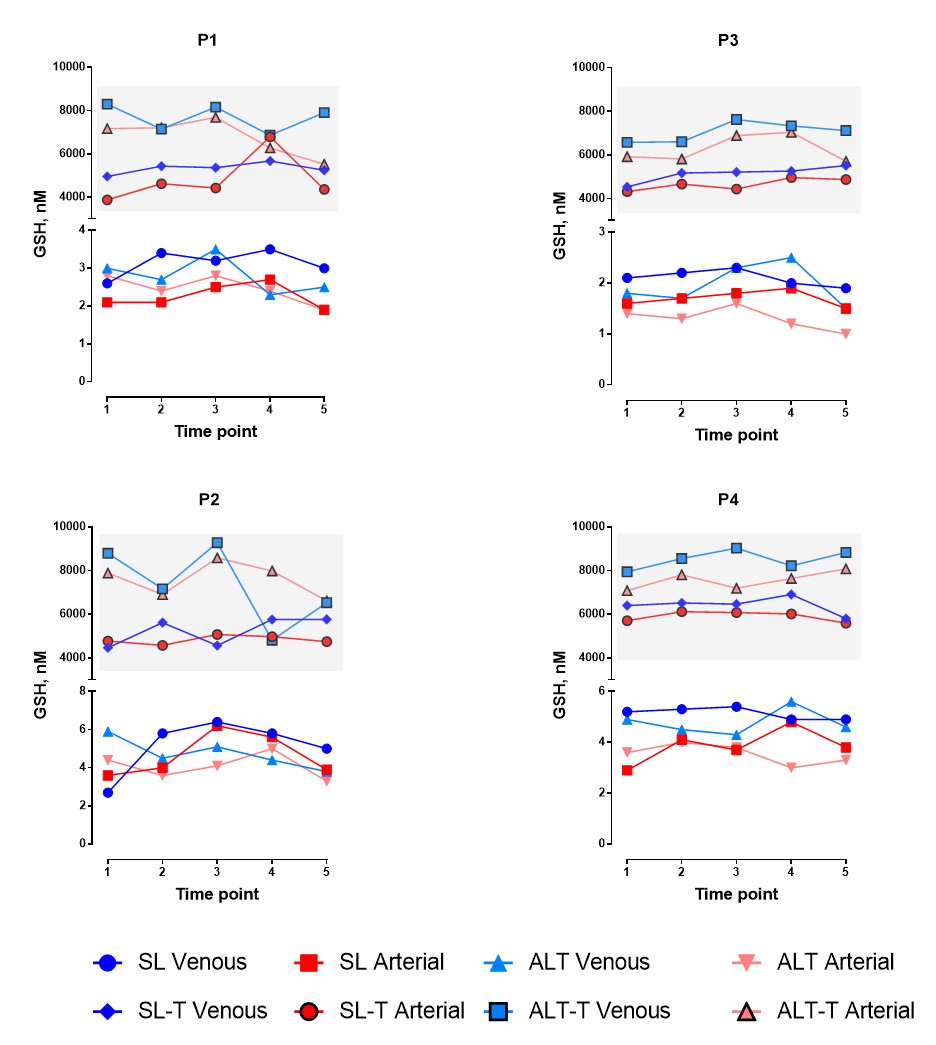


**Suppl. Fig.2:** Arterial (red) and venous (blue) steady-state concentrations of free and total (T) homocysteine (HCys) in study participants (P1, P3 male, P2, P4 female) at rest (T1), during exercise (T2-4) and in early recovery (T5) at sea level (SL; circles and squares) and at high altitude (ALT – light triangles).


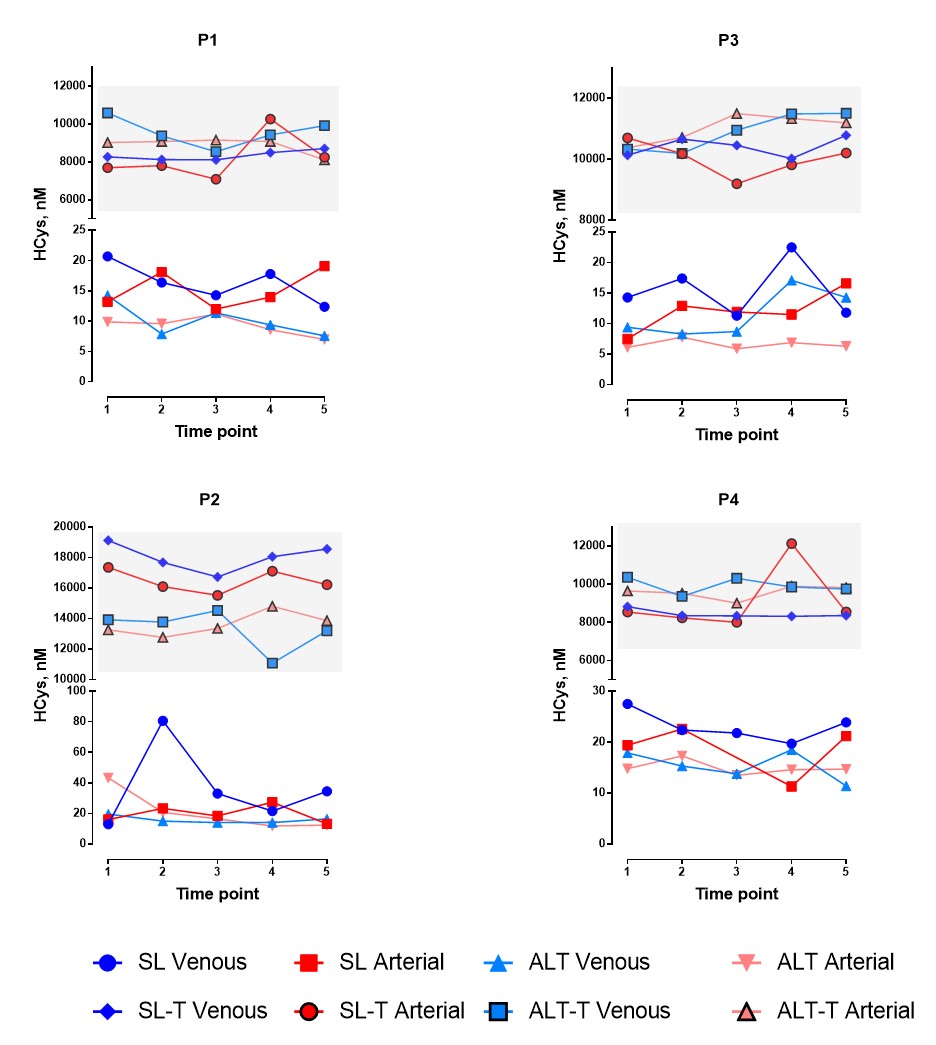


**Suppl. Fig.3:** Arterial (red) and venous (blue) steady-state concentrations of free and total (T) N-acetylcysteine (NAC) in study participants (P1, P3 male, P2, P4 female) at rest (T1), during exercise (T2-4) and in early recovery (T5) at sea level (SL; circles and squares) and at high altitude (ALT – light triangles).


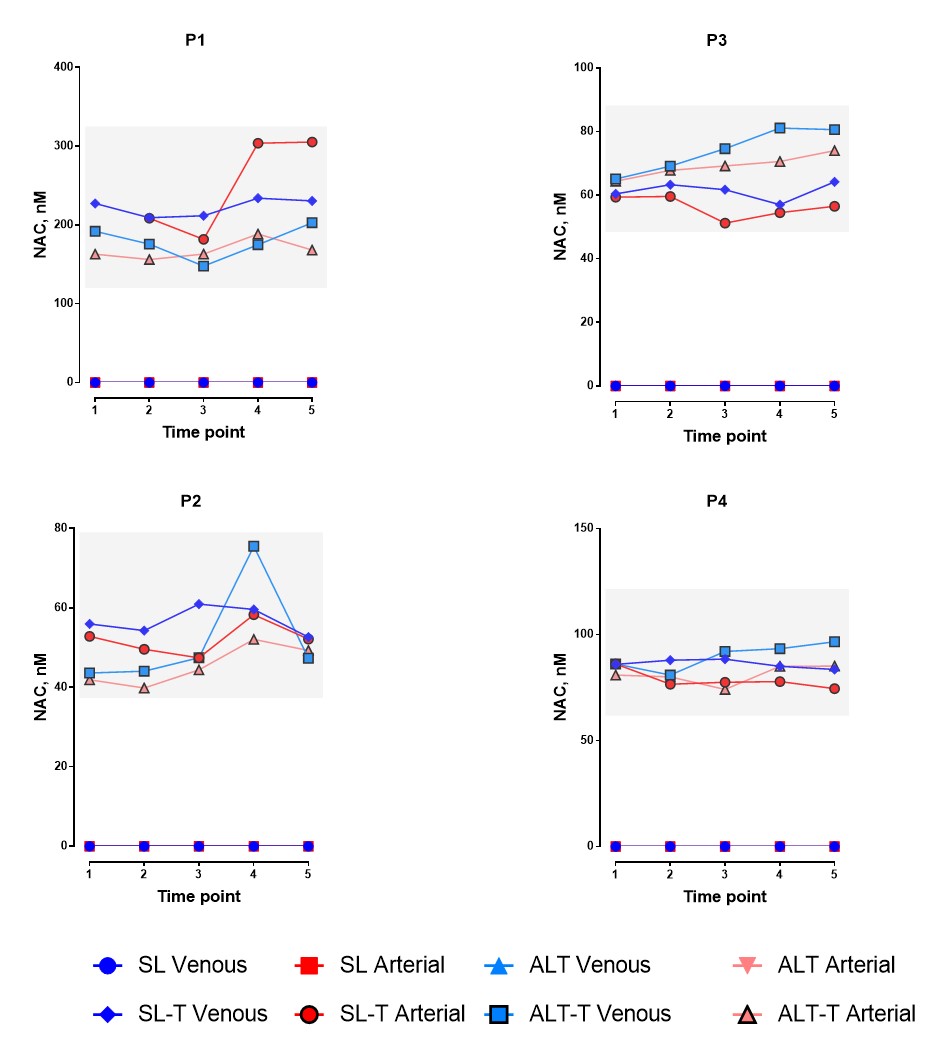


**Suppl. Fig.4:** Arterial (red) and venous (blue) steady-state concentrations of free and total (T) cysteinylglycine (CysGly) in study participants (P1, P3 male, P2, P4 female) at rest (T1), during exercise (T2-4) and in early recovery (T5) at sea level (SL; circles and squares) and at high altitude (ALT – light triangles).


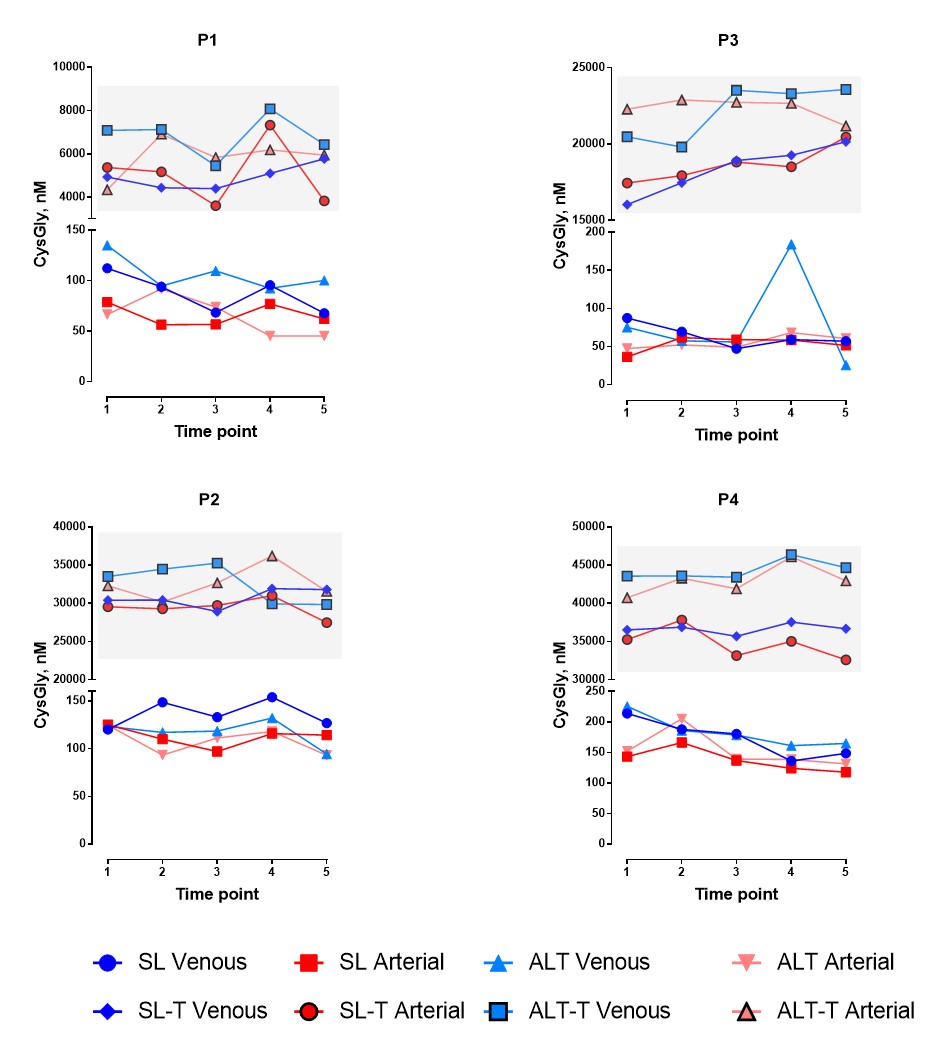


**Suppl. Fig.5:** Arterial (red) and venous (blue) steady-state concentrations of free and total (T) glutamylcysteine (GluCys) in study participants (P1, P3 male, P2, P4 female) at rest (T1), during exercise (T2-4) and in early recovery (T5) at sea level (SL; circles and squares) and at high altitude (ALT – light triangles).


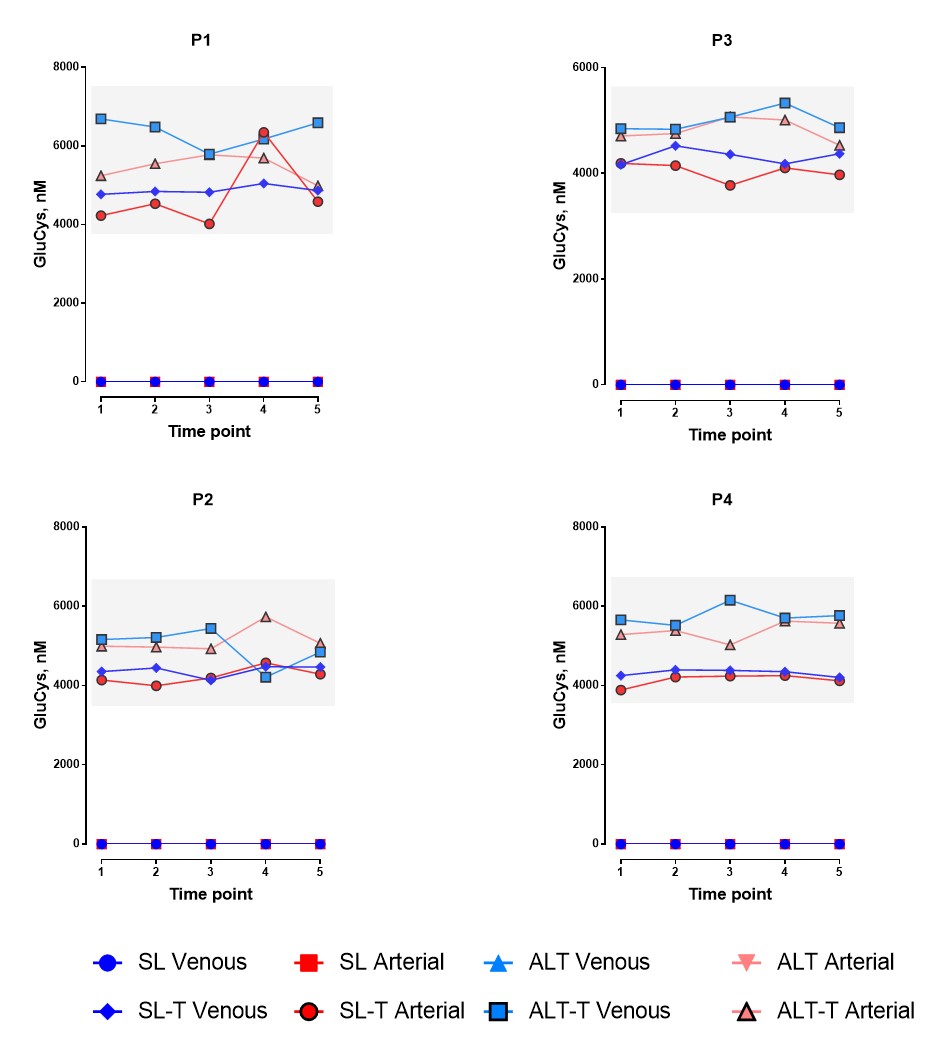


**Suppl. Fig.6:** Arterial (red) and venous (blue) steady-state concentrations of total free thiols (TFT) in study participants’ (P1, P3 male, P2, P4 female) plasma at rest (T1), during exercise (T2-4) and in early recovery (T5), at sea level (SL; circles and squares) and at high altitude (ALT – light triangles).
